# Classification of Targets and Distractors in an Audiovisual Attention Task Based on Electroencephalography

**DOI:** 10.3390/s23239588

**Published:** 2023-12-03

**Authors:** Steven Mortier, Renata Turkeš, Jorg De Winne, Wannes Van Ransbeeck, Dick Botteldooren, Paul Devos, Steven Latré, Marc Leman, Tim Verdonck

**Affiliations:** 1 IDLab—Department of Computer Science, University of Antwerp—imec, Sint-Pietersvliet 7, 2000 Antwerp, Belgium; renata.turkes@uantwerpen.be (R.T.); steven.latre@uantwerpen.be (S.L.); 2 WAVES Research Group, Department of Information Technology, Ghent University, 4 Technologiepark 126, Zwijnaarde, 9052 Ghent, Belgium; jorg.dewinne@ugent.be (J.D.W.); wannes.vanransbeeck@ugent.be (W.V.R.); dick.botteldooren@ugent.be (D.B.); p.devos@ugent.be (P.D.); 3 Department of Art, Music and Theater Studies, Institute for Psychoacoustics and Electronic Music (IPEM), Ghent University, 9000 Ghent, Belgium; marc.leman@ugent.be; 4 Department of Mathematics, University of Antwerp—imec, Middelheimlaan 1, 2000 Antwerp, Belgium; tim.verdonck@uantwerpen.be

**Keywords:** electroencephalography, machine learning, classification, P300, event-related potential

## Abstract

Within the broader context of improving interactions between artificial intelligence and humans, the question has arisen regarding whether auditory and rhythmic support could increase attention for visual stimuli that do not stand out clearly from an information stream. To this end, we designed an experiment inspired by pip-and-pop but more appropriate for eliciting attention and P3a-event-related potentials (ERPs). In this study, the aim was to distinguish between targets and distractors based on the subject’s electroencephalography (EEG) data. We achieved this objective by employing different machine learning (ML) methods for both individual-subject (IS) and cross-subject (CS) models. Finally, we investigated which EEG channels and time points were used by the model to make its predictions using saliency maps. We were able to successfully perform the aforementioned classification task for both the IS and CS scenarios, reaching classification accuracies up to 76%. In accordance with the literature, the model primarily used the parietal–occipital electrodes between 200 ms and 300 ms after the stimulus to make its prediction. The findings from this research contribute to the development of more effective P300-based brain–computer interfaces. Furthermore, they validate the EEG data collected in our experiment.

## 1. Introduction

The interaction between humans and artificial intelligence (AI) still lacks the level of engagement and synchronization that symbolizes the interactions between humans. The primary goal of the WithMe project (The WithMe project is a research project funded by the Research Foundation Flanders (FWO). More information can be found at https://researchportal.be/en/project/withme-making-human-artificial-intelligence-interactions-more-entraining-and-engaging, accessed on 1 November 2023.) is to thoroughly study the processes that occur in the human brain during joint activities with another individual, such as working towards shared objectives [1]. The brain signals collected in this study were primarily indicative of attention but also of emotion and reward. The purpose of this research was to determine relevant electroencephalography (EEG) features indicative of attention using machine learning (ML).

To this end, a specific experiment was designed. Temporal audiovisual integration and support of visual attention by sound was well demonstrated in the pip-and-pop experiment [2]. The pip-and-pop experiment is based on a visual search, which does not lead to a strong visually evoked potential. Moreover, as we expected that the rhythmic presentation of target stimuli also affects working memory, the task was replaced with a modified digit-span task, where five target digits had to be remembered and reported in our experiment [1]. This task involves visual attention, working memory, and sequence recall. To investigate the role of attention, we directly measured the brain activation by means of EEG. Specifically, event-related potentials (ERPs) have been shown to be excellent tools for studying attention [3,4]. Risto Näätänen was a pioneer in this domain, as they studied the connection between ERPs and attention, which led to the discovery of (auditory) mismatch negativity ERP [5,6,7,8]. Additionally, research has shown that the amplitude of P300 is directly related to the amount of attentional resources available for stimulus processing [8,9,10,11]. The P300 ERP is observed to be elicited for deviant stimuli in a sequence of standard stimuli, where the deviant stimuli are in some way more relevant to the presented task [12,13,14]. In our experiment, we thus expected that the targets would elicit a P300 ERP. Research showed that the P300 actually consists of two subcomponents: P3a and P3b [15]. P3a generally reaches its peak around 250 ms to 280 ms after a stimulus and is associated with attention-related brain activity [16]. On the other hand, the P3b peak can vary in latency, lying between 300 ms and 500 ms post-stimulus [15]. P3b is elicited by improbable events, provided that the improbable event is somehow relevant to the task at hand [17]. In our experimental setting, we expected to elicit a P3a, as the target stimuli were not scarce (there are approximately 50% targets and 50% distractors), and our experiment was designed to evoke attention. We did not expect to elicit a P3a for distractors, as subjects should not pay attention to them.

The goal of this work was to accurately classify whether a target or distractor stimulus was presented to the subject based on the subject’s EEG data. For this purpose, we applied different existing ML methods to classify EEG data and investigate which method performed best on our specific use case. As we expected to elicit attention when a target was shown (and not when a distractor was shown), the trained ML was effectively an attention detector. We expected the attention to manifest itself in the form of a P3a ERP, and therefore we expected that the model would base its predictions on the presence of a P3a peak. Detecting P3a signals and, more broadly, P300 signals has a wide range of applications [18,19], particularly in P300-based brain computer interfaces (BCIs) [20], for example, in spellers [21,22,23] and intelligent home control systems [24,25]. These applications can be of great help for patients suffering from amyotrophic lateral sclerosis (ALS) or spinocerebellar ataxia, as they can enable them to communicate in a daily environment [21,23,26,27]. In the literature, a wide array of techniques have been reported for classifying and detecting P300 [28]. Some techniques rely on a data transformation and subsequently use logistic regression to classify the transformed data, for example, xDAWN + RG [29,30,31,32]. Recently, deep learning approaches, primarily based on convolutional neural networks (CNNs), for example, EEGNet [33,34,35], have also gained in popularity [36,37,38]. Finally, as EEG data are essentially heavily correlated multivariate time series, it is possible to apply standard time series classification techniques as well [39,40,41].

Building BCIs that are trained on multiple subjects and generalize well to previously unseen subjects holds significant value [42]. Indeed, BCIs often need to be retrained or at least calibrated for the end user [43], which is a costly and user-unfriendly process [44,45]. However, due to intersubject variability in EEG data, training models that generalize to multiple-subjects (cross-subject (CS) models) is a harder task than training models for one-subject (individual subject (IS) models) [44,45,46]. For this reason, we also investigated the hypothesized drop in performance when transitioning from IS to CS models. Additionally, the ML models should be able to make predictions in real time, as this is essential in real-world BCI applications.

Finally, we analyzed which EEG channels and time points were used by our models to make its predictions and checked whether these align with the expected P3a attention signature. However, ML models such as CNNs are considered “black boxes”, as no clear explanation exists for the decisions made by these models [47]. The rapidly emerging and improving field of explainable AI (xAI) aims to tackle these issues by providing insights into ML models’ decision-making processes. Some xAI techniques that are often used to gain insights into EEG classification models are local interpretable model-agnostic explanations (LIME) [48,49], DeepLIFT [33,50,51], and saliency maps [52,53,54], among others.

In summary, we aimed to enhance the interaction between humans and AI and designed a novel experiment for this purpose. Specifically, we considered building a ML model to recognize targets shown to a subject, which equates to creating an attention detector. These models should ideally generalize well to previously unseen subjects. The primary contributions of this study are:

- Training of state-of-the-art classification methods to accurately predict target and distractor stimuli based on EEG data.
- Analysis of the performance difference between IS and CS models.
- Investigation into which EEG channels and time points were important for the model predictions, using xAI.

Ultimately, the contributions of this research collectively advance our understanding of human–AI interaction and will aid in the development of more effective BCIs and their associated applications.

The remainder of this paper is structured as follows: Section 2.1 introduces the WithMe experiment and dataset, while Section 2.2 explains the data preprocessing routine. Section 2.3 illustrates the classification problems and provides a description of the classification methods used in this study. Section 3 presents the results and provides an in-depth analysis of the best-performing model. This section also contains an extensive discussion of the achieved results. Finally, in Section 4, we draw conclusions and provide possible directions for future research.

## 2. Materials and Methods

In this section, we describe the WithMe dataset that was analyzed using acMLin this study. We then define the preprocessing steps that were applied to the EEG data. Finally, we present the classification problem and the classification methods and metrics that we used to tackle this problem.

### 2.1. The WithMe Experiment

A total of 42 young adults participated in the experiment (21 women, 21 men; mean age 23.64±2.69 years). They were recruited through the university network and through the social and professional networks of the authors. All subjects declared to have normal or corrected to normal vision and showed normal hearing (<25 dB hearing loss) for the frequencies used in the experiment based on a standard pure tone audiometry hearing test. To mitigate the potential influence of age and/or intelligence, the subject’s age range was limited to young adults under 30 years, and they were only accepted if they were enrolled in or had finished some form of higher education.

Before starting the experiment, subjects had to fill in a questionnaire that asked for general background information to identify some personal characteristics. For example, subjects were asked if they ever enrolled in some form of musical education and/or were an active musician. More details about the questionnaires and their extensive analyses are described in [1].

**Figure 1:**
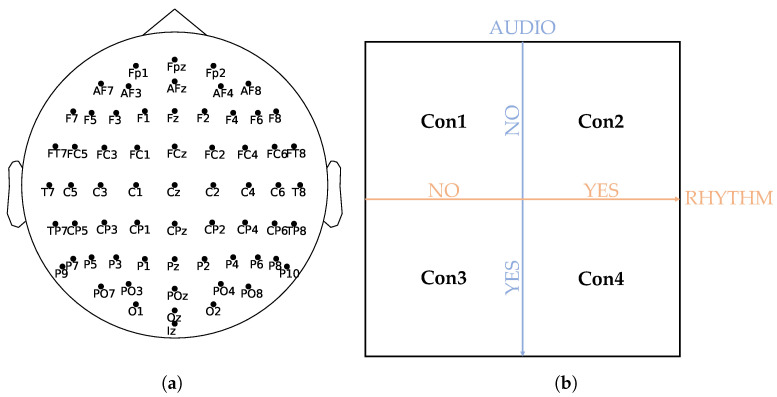
(**a**) The WithMe experiment used 64 electrodes, organized according to the 10-10 system. Figure made using [55]. (**b**) Depending on the condition, the subject received no support (Con1), visual rhythmic support (Con2), auditory nonrhythmic support (Con3), or visual rhythmic and auditory support (Con4).

The experiment consisted of a modified digit-span task. A target digit was presented, followed by either no, one, or two distractor digits; another target; etc. One sequence of digits always consisted of five targets and five distractors, although the subjects did not know this a priori. After one sequence of targets and distractors was presented, the subject had to report all targets in the correct order that they were presented to them. The targets and distractors were presented as an encircled number x∈{0,1,2,⋯,9}. Additionally, a distractor could show up as an empty circle. Target digits were colored black (rgb(0,0,0)), while distractor digits were displayed in dark gray (rgb(x,x,x),x∈[50,75]), with the exact value of *x* determined individually to ensure the difference between targets and distractors was just noticeable. An example sequence is shown in Table 1. In total, 30 different sequences of targets and distractors were created, which were shown to the subject under four different conditions in a pseudo-randomized order [1]. Depending on the condition, the subject received either no support (Con1), visual rhythmic support (Con2), auditory nonrhythmic support (Con3) or visual rhythmic and auditory support (Con4), as shown in Figure 1b. This added up to a total of 120 sequences shown to the subject. In conditions with auditory support (Con3 and Con4), targets were accompanied by a 500 Hz tone burst, which lasted 50 ms. In conditions with rhythmic support, targets were presented with a fixed time interval of exactly 1.25 s between them. In these rhythmic conditions, the sequence of digits was preceded by five rhythms inducing stimuli to induce the subject with the rhythm. In Con2, this was achieved using empty black circles, while in Con4 auditory, tone bursts were used to induce the rhythm. For more detailed information about the experiment, we refer the reader to the original paper that describes the experiment and behavioral analysis [1].

### 2.2. Dataset and Preprocessing

During this experiment, EEG data were sampled at 2048 Hz using the standard 64-electrode EEG 10-10 system, as shown in Figure 1a. Thereafter, standard EEG preprocessing techniques were applied. The data were re-referenced to the average of both earlobes, just one earlobe if the other one was too noisy, or another pair of channels if both earlobes were badly recorded or too noisy. In the case of bad channels, these were identified and removed. EEG data were notch-filtered at the line frequency (50 Hz) and its multiples, after which a bandpass filter from 0.2 Hz to 100 Hz was applied. The data were split into epochs, ranging from 0.2 s prestimulus to 1.0 s poststimulus, resulting in 1.2 s epochs. Independent component analysis (ICA) was applied to the epoched data. Any components that represented artifacts were removed through visual inspection of the ICA components.

During the previous steps, some channels were marked as bad channels. Instead of dropping these channels, we chose to interpolate them using their neighboring channels, as the former would result in an inconsistent number of channels across sequences and subjects. The interpolation was performed using the MNE-Python package (All Python packages that were used in this work can be found in Table A1, together with their version number and citation.) [55]. Finally, the data were downsampled to 50 Hz, as this reduced computation time, decreased file read/write time, and saved memory, while generally leading to little or no loss of information [56]. We should however note that, based on the Nyquist theorem, this limits the highest frequency that can be accurately represented to half of the sampling frequency, i.e., 25 Hz. This preprocessing routine ideally resulted in 600 target epochs, 600 distractor epochs, and 300 induction epochs for each of the 42 subjects. However, during preprocessing, some epochs were rejected for various reasons, for example, an excessive number of bad electrodes or too much noise. On average, less than 0.6% of the epochs were rejected per subject.

**Figure 2:**
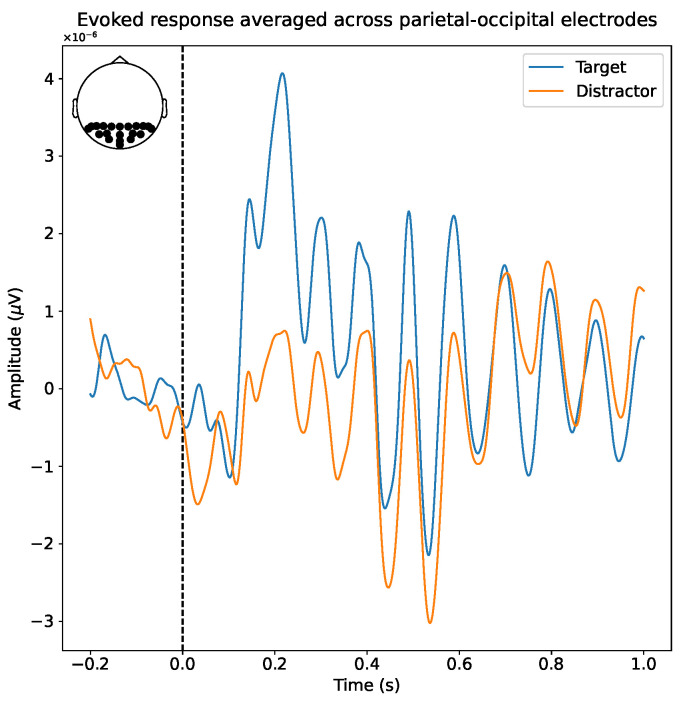
The evoked response for targets and distractors for one subject. The data were averaged over all electrodes of the parietal-occipital region in the brain, as indicated in the figure inset.

As mentioned in Section 1, we expected to observe a P3a ERP when subjects saw a target stimulus. The P3a ERP is characterized by a positive voltage deflection between 250 ms and 280 ms after the stimulus, although the exact timing can vary [16,57,58]. As our experiment used visual stimuli, we expected the P3a ERP to be the most pronounced in the parietal-occipital region of the brain [56]. Figure 2 shows the evoked response for one subject, averaged over all parietal-occipital electrodes, as indicated in the figure inset. We observed a clear positive deflection between 200 ms and 300 ms after the stimulus, in line with our expectations.

### 2.3. Classification Problem

The models trained in this study considered a two-class classification problem (target versus distractor) and took single-trial EEG epochs as input to predict a binary label. As the data were downsampled to 50 Hz, one epoch contained 60 time steps, for 64 electrodes. This means that the input was of shape (N,64,60), with *N* being the number of epochs. It is important to note that it was impossible to obtain 100% accuracy for this model. Indeed, the model made a prediction based on the subject’s assessment of a stimulus, and it is possible that a subject did not correctly recognize all targets and distractors. As the ground truth labels were based on the predefined labels of the experiment, it is possible that there was a slight mismatch between the labels and the subject’s perceived class. Nevertheless, we assumed that this problem was rare, meaning that commonly used metrics, for example, accuracy, provided a valid interpretation.

Ideally, the models should be able to generalize to previously unseen subjects. To investigate this, we trained the models in two ways: models trained on IS and models trained on (nearly) all subjects, also called CS models. The former was evaluated using a randomly sampled test set with a standard 80% train and 20% test set split, while the latter were evaluated using a leave-one-out (LOO) methodology. In general, models perform better when trained and tested on individual subjects [59]. This can be attributed to the variability in subject’s EEG data elicited by the same stimuli. However, in practice, EEG classification models should ideally extrapolate to previously unseen subjects. For example, BCIs often need to be calibrated for new end users, which usually takes 20 to 30 min [60,61,62]. Therefore, it is interesting to investigate which model architectures are best suited to build subject-independent classifiers.

#### 2.3.1. Classifiers

To solve this classification problem, we trained and evaluated different existing ML models. Different methodologies for classifying EEG data exist. For example, we can extract features from EEG data and use these extracted features as the input to a classifier. These features can, among others, be extracted from the time domain, frequency domain, or the time–frequency domain, or through methods such as principal component analysis [63,64]. Such methods are referred to as feature-based methods. Another common approach uses raw or preprocessed EEG data as the input to the classifier. In this approach, commonly referred to as end-to-end methods, the classifier itself extracts relevant features from the data during training and uses these features to classify a sample. As both methodologies are interesting approaches, we used methods belonging to both approaches. In this study, we applied four distinct classifiers and compared the results on a novel data set. An overview of the classifiers and their methodologies is presented in Table 2. First, we applied the xDAWN pipeline, which has demonstrated significant success in several EEG classification tasks [29,30]. For example, the BCI challenge organized as part of the IEEE Neural Engineering Conference 2015 was won by an xDAWN-based approach [30]. In this study, we employed a similar approach, consisting of first estimating two sets of xDAWN spatial filters, one for each class (target and distractor) [29]. Subsequently, the grand average evoked potential of each class iwass filtered using the corresponding filters, after which they were concatenated to each of the trials. Then, the covariance matrix of each resulting trial was used as a feature for the next steps in the pipeline [65,66]. The next step was to project the covariance matrices on the tangent space using a Riemannian metric, as described in [31,32]. After these feature extraction steps, a classifier was used to make the final predictions. Based on [30,67], we used logistic regression [68]. For the remainder of this paper, we refer to this method as xDAWN + RG (xDAWN + Riemannian Geometry). Calculating the xDAWN covariance matrices and projection to the tangent space were performed using the PyRiemann package [67].

The second method we used was EEGNet [33]. EEGNet exhibits strong performance on a variety of EEG-based classification tasks, such as P300 ERP classification [33,53] and motor imagery classification [69]. Whereas the previous method used extracted features as the input to the classifier, EEGNet performs both the feature extraction and classification. EEGNet is a deep learning model, more specifically, a CNN. As its name suggests, EEGNet is optimized for classifying EEG data by employing a set of specific design choices. First, it uses temporal convolutions to learn frequency filters [33]. As suggested by the authors, the length of the temporal kernel used in these convolutions is set to half the sampling rate, which allows the model to capture frequency information at frequencies of 2 Hz and higher [33]. Second, depthwise convolutions are used to learn frequency-specific spatial filters. In this context, depthwise convolutions have two main advantages. First, they noticeably reduce the number of trainable parameters, since these convolutions are not fully connected to the previous layer; instead, they are connected to each feature map individually. This induces the second, EEG-specific advantage: the model learns spatial filters for each temporal filter, which enables the efficient extraction of frequency-specific spatial filters [33]. The last convolutional part consists of a separable convolution, which is a combination of depthwise and pointwise convolution. The former learns how to summarize individual feature maps in time, while the latter learns how to optimally combine the feature maps [33]. Finally, all features are passed to a dense layer for classification. More details on the EEGNet architecture can be found in [33]. We used the standard EEGNet-8,2 layout, which means that the model learns 8 temporal filters and 2 spatial filters per temporal filter.

The first two methods were designed for EEG specifically. However, since EEG data are essentially heavily correlated multivariate time series, it was interesting to study the results of a more general method designed to classify such time series. To this end, we applied random convolutional kernel transform (Rocket) [39]. Based on the success of CNNs for time series classification, Rocket uses random convolutional kernels combined with simple linear classifiers. This novel combination achieved state-of-the-art performance on the UCR time series archive using only a fraction of the computational cost of existing methods [39,70]. As a follow-up to Rocket, the authors also designed MiniRocket [40]. They claimed that MiniRocket can be trained up to 75 times faster than Rocket, while achieving nearly the same performance. MiniRocket distinguishes itself from Rocket primarily by reducing the degree of randomness that Rocket generates, resulting in MiniRocket being almost deterministic [40]. Since methods used to classify EEG data, such as EEGNet, can be very computationally expensive, it was worth exploring the effectiveness of less computationally expensive methods. We used the Rocket and MiniRocket implementations in the sktime package and combined them with the ridge regression classifier implemented in scikit-learn, as suggested by the authors [39,40,68,71].

#### 2.3.2. Metrics

To allow the comparison of various approaches, it is essential to have predetermined performance metrics. We focused on three metrics that are widely used in the EEG classification literature: accuracy, F1 score, and area under the receiver operating characteristic curve (ROC AUC) [72]. First, the accuracy states the number of correctly classified samples across both classes. Second, the F1 score assesses the predictive performance of a model via calculating the harmonic mean of the precision and recall metrics. The equations used to calculate the accuracy and F1 score are given in Equations (1) and (4), respectively, where we use the following abbreviations: true positive (TP), false positive (FP), true negative (TN), and false negative (FN). Third, by plotting the true positive rate against the false positive rate for different classification thresholds, we obtained the ROC curve. The ROC AUC is defined as the area under this curve and provides a measure for how well a classifier can distinguish between true and false samples or, in our case, targets and distractors, respectively. Finally, we also assessed the required training time and model complexity of all models.

(1) $$\mathrm{accuracy}=\frac{\mathrm{TP}+\mathrm{TN}}{\mathrm{TP}+\mathrm{TN}+\mathrm{FP}+\mathrm{FN}}$$

(2) $$\mathrm{precision}=\frac{\mathrm{TP}}{\mathrm{TP}+\mathrm{FP}}$$

(3) $$\mathrm{recall}=\frac{\mathrm{TP}}{\mathrm{TP}+\mathrm{FN}}$$

(4) $$F1-\mathrm{score}=2\cdot \frac{\mathrm{precision}\times \mathrm{recall}}{\mathrm{precision}+\mathrm{recall}}=\frac{2\mathrm{TP}}{2\mathrm{TP}+\mathrm{FP}+\mathrm{FN}}$$

## 3. Results and Discussion

### 3.1. Individual Subject Models

**Figure 3:**
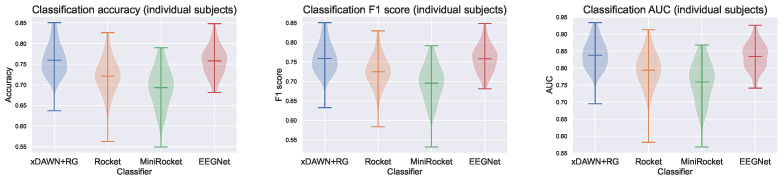
Violin plots of the test accuracy, F1 score, and AUC for models trained on individual subjects.

The performance of the models, assessed using the metrics introduced in Section 2.3.2, is shown in Table 3 and Figure 3. Using an EEG-specific model architecture benefits the performance of IS models. While xDAWN + RG and EEGNet perform equally well, they demonstrate superior accuracy, F1 score, and area under the curve (AUC) in comparison to MiniRocket and Rocket. As expected, MiniRocket achieves slightly inferior performance compared to Rocket. However, MiniRocket’s training time was 15 times faster on our dataset. Notably, while xDAWN + RG and EEGNet exhibit equal performance, xDAWN + RG is significantly less computationally expensive than EEGNet. On central processing units (CPUs) alone, EEGNet’s training time is nine times longer. Although training times can be accelerated for EEGNet using (expensive) graphics processing units (GPUs), even when using an NVIDIA GTX 1080 GPU, EEGNet still requires 2.5 times as long to train as xDAWN + RG.

### 3.2. Cross-Subject Models

**Figure 4:**
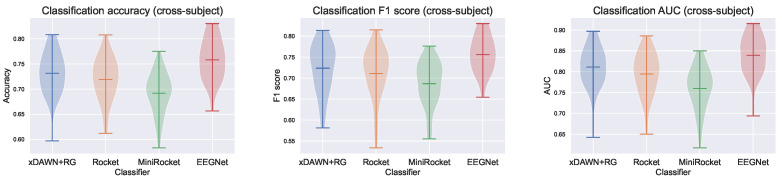
Violin plots of the test accuracy, F1 score, and AUC for cross-subject models.

Similar results were obtained for the CS models, where EEG-specific approaches perform better than Rocket and MiniRocket, as shown in Table 4 and Figure 4. However, in this scenario, EEGNet outperforms xDAWN + RG. We hypothesized that this can be attributed to EEGNet’s added complexity and its greater number of parameters compared to xDAWN + RG. This additional capacity is more likely to be able to learn features that extrapolate well to previously unseen data points.

### 3.3. Individual-Subject Models vs. Cross-Subject Models

**Figure 5:**
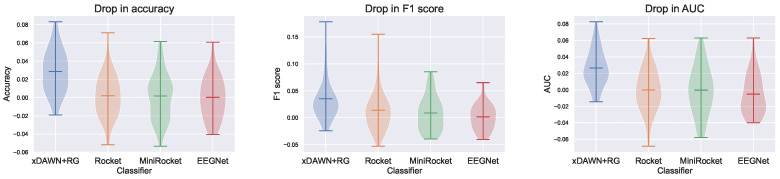
Violin plots of the drop in performance, calculated by subtracting the test performance of cross-subject models from that of individual subject models.

As we discussed in Section 2.3, we expected the performance of the IS models to be better than that of the CS models. Despite having access to a significantly larger amount of data, constructing a CS model is a considerably more challenging task. To illustrate the performance disparity between the two, refer to Table 5 and Figure 5, which showcase the performance difference by subtracting the CS model’s performance from that of the IS model. EEGNet, MiniRocket, and Rocket exhibit similar performance for both IS and CS models. However, the xDAWN + RG model demonstrates a noticeable decrease in performance. Given the lower absolute performance of the (Mini)Rocket models compared to EEGNet and xDAWN + RG, we focus on the latter for the remainder of this discussion. We hypothesize that the inferior performance on CS models when using xDAWN + RG can be attributed to its simpler and lightweight nature. Furthermore, xDAWN + RG works by first calculating the evoked responses for all classes. These can differ significantly from subject to subject, both in P3a peak height and in time [27,73,74]. The convolutional nature of EEGNet likely enables it to capture the temporal dynamics of the elicited responses more effectively across different subjects. It is important to note that the CS models have access to a significantly larger corpus of training data than the IS models, which is part of the reason that they keep up reasonably well with the IS models.

### 3.4. Analysis of the EEGNet Cross-Subject Model

We then conducted further investigation into the CS EEGNet model. We conducted this analysis for the EEGNet model, as it performed the best in both the IS and CS scenarios. Furthermore, we included this analysis only for the CS models, as they are the most useful in practice due to their generalization capabilities. However, the conclusions are similar for the IS models.

#### 3.4.1. Confusion Matrices

**Figure 6:**
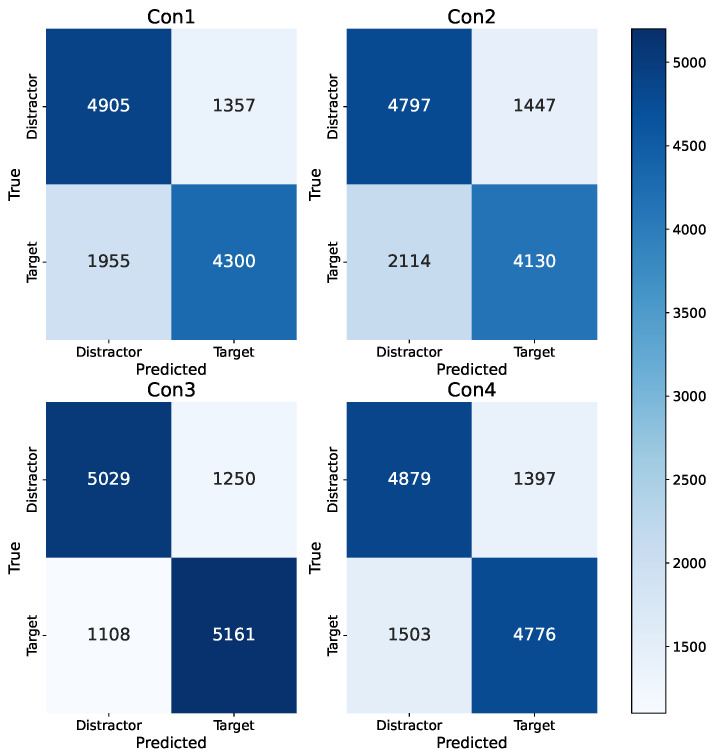
Confusion matrices for the cross-subject EEGNet model, split across the four conditions defined in Figure 1b. The confusion matrices were obtained by aggregating all the test predictions of the CS models.

First, we investigated whether the model focused on the correct features to make a prediction. For example, it is possible that we trained a sound detector instead of a target/distractor model. Indeed, conditions Con3 and Con4 contained auditory clues for the target. Theoretically, the model could rely solely on the activation in the auditory stimuli processing region of the brain and achieve acceptable performance. For example, if the model performs perfectly on Con3 and Con4, while predicting all trials belonging to Con1 and Con2 to be distractors (due to the absence of auditory stimuli), it would achieve an accuracy of approximately 75%. The confusion matrices in Figure 6 negate this assumption. The model performs comparably in detecting distractors under all conditions. However, the model performs slightly better at identifying targets correctly for Con3 and Con4. The accuracies for specific conditions, shown in Table 6, also reflect this. Indeed, the accuracies for conditions Con3 and Con4 are higher than the accuracies for Con1 and Con2. We hypothesized that the inclusion of auditory support causes an additional signature in the EEG data, making it easier for the model to recognize targets. Additionally, it was already confirmed through a previous analysis that the subjects were able to recall the targets better in conditions with auditory support [1].

#### 3.4.2. Saliency Maps

**Figure 7:**
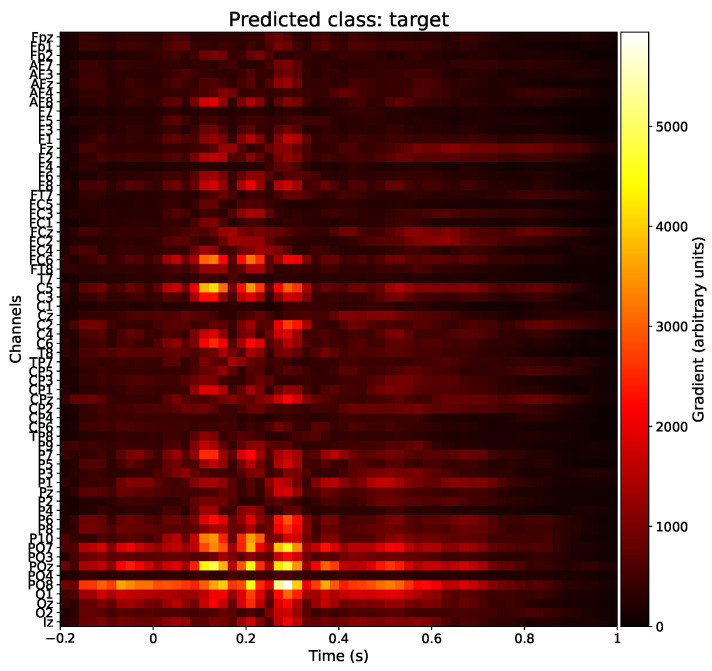
Saliency map for epochs labeled as targets using the cross-subject EEGNet model. We averaged normalized saliency maps over all 42 test subjects for the CS model.

**Figure 8:**
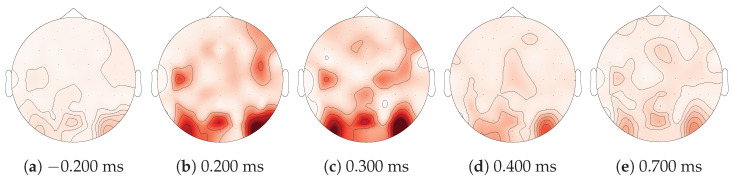
The saliency map from Figure 7, shown as a topographic map at five different times. A deeper shade of red indicates a larger gradient. At t=−0.200 ms and t=0.700 ms, the gradients are near zero, indicating that the model does not use these timings. Contrary, for t∈{0.200,0.300,0.400} ms, there is a large gradient in the parietal-occipital region of the brain.

Next, we explored the electrodes and timings that are predominantly used by our models for making predictions. Trivially, we expected that the model would not use the prestimulus (t<0) EEG data. As deep learning methods such as EEGNet are inherently black box models, we resorted to xAI methods to obtain (interpretable) insights into the model. A possible technique is a saliency map, which is a visual representation that highlights the degree of importance of regions or features in an input sample in the model prediction [52]. To generate a saliency map, the gradient of the model output with respect to the input sample is computed using backpropagation [53]. More specifically, this process involves fixing the weights of the trained model and propagating the gradient with respect to the layer’s inputs back to the first layer that receives the input data. Figure 7 shows such a saliency map. This saliency map illustrates the electrodes and timings that had the greatest average impact on the model prediction when identifying a sample as a target. It was computed by first calculating the average saliency map for each test subject individually, then normalizing these saliency maps, and ultimately taking the average across all 42 subjects. In Figure 8, the same information is repeated, displayed as a topographic map at five time points. From Figure 7 and Figure 8, we can see that our model predominantly used the parietal-occipital electrodes and time points between 200 ms and 300 ms after the stimulus to make its prediction, which is what we expected. We also investigated the saliency maps under different conditions but noticed no significant difference between the conditions.

## 4. Conclusions and Future Work

The WithMe project has led to the collection of a large, novel EEG dataset that can be used to create ML methods to automatically detect attention using P3a ERPs in single-trial data. This is of great importance to BCIs, as they often rely on the P3a, r, more broadly, the P300 ERP and have a wide range of applications.

We successfully achieved the goal in this study, which was to classify target and distractor stimuli based on the subject’s EEG data. To achieve this goal, we studied four classification methods that differed significantly in origin and complexity. We investigated the performance of these methods both as IS and CS models, with the latter being the most practically relevant due to its generalization capabilities. For the IS models, xDAWN + RG and EEGNet obtained an accuracy of 76%, outperforming MiniRocket and Rocket. While EEGNet was able to obtain the same accuracy of 76% in the CS case, the accuracy of xDAWN + RG dropped to 0.73%. We attribute this difference to the larger complexity of EEGNet, which likely enables it to generalize better to previously unseen subjects. The drop in performance between IS and CS models was not as pronounced as we expected it to be and was even nonexistent for EEGNet. We attributed this to the fact that the CS models had approximately 42 times more training data available. The EEGNet CS model performed slightly better on samples recorded under conditions Con3 and Con4, which were the conditions that included auditory support. While EEGNet achieved the best performance overall, it also had the highest model complexity (highest number of trainable parameters) and took the longest time and most computing resources to train. However, all four models were able to make predictions in real time. This property is essential for real-world human–AI interaction experiments and applications.

Finally, the application of xAI enabled us to investigate which EEG channels and time points were used by the otherwise black box EEGNet CS model to make its predictions. Indeed, using saliency maps, we concluded that the model primarily based its prediction on the values of the electrodes in the parietal-occipital region between 200 ms and 300 ms after the stimulus. This is in line with our hypotheses, as we expected to elicit an attention-related P3a ERP in the parietal-occipital region of the brain when the subject saw a target digit.

In conclusion, we achieved the goal of accurately classifying targets and distractors based on a subject’s EEG data. At the same time, our work contributes to the development of more effective BCIs and their applications. Finally, we validated the EEG data collected in the WithMe experiment.

While this study provides valuable insights into attention detection using EEG data, it is important to acknowledge some limitations. For example, as mentioned in Section 2.3, part of the data used to train the model were labeled incorrectly, as the ground truth labels were based on the predefined labels of the experiment rather than the subject’s perceived class. A possible solution is to limit the data to samples where the entire sequence is reported correctly. However, this means that we would lose a lot of data, which would in turn decrease the performance of the models. Alternatively, we could remove all “bad sequences”, where a bad sequence is defined as a sequence in which none of the targets were remembered correctly. This could be caused by either incorrectly identifying the stimuli or by bad memory management, despite correctly identifying the targets and distractors. However, the number of answers that did not include at least one of the target digits (regardless of its place in the sequence) is negligible.

In future work, an experiment dedicated to attention should be used to circumvent the limitations regarding bad labels, as described in Section 4. This would allow for labels that exactly correspond to the subject’s perception of a stimulus, which would in turn lead to more accurate attention detectors. The ultimate goal could then be to use this attention detector in a BCI to detect whether a subject paid attention. In case they did not, the BCI could repeat the sequence or stimulus to make sure that the subject can act accordingly. This could also improve learning systems, that is, systems that know whether a student actually paid attention to the provided information [75,76]. Regarding the training and optimization of ML models, it would be interesting to include an exhaustive feature selection procedure to allow the ML model to focus on the (most) relevant features. Additionally, we want to explore other ways to enable CS generalization, for example, using transfer learning [77,78]. This could further increase the generalization performance of all methods. In particular, this has the potential to elevate the performance of lightweight models such as xDAWN + RG to that of the computationally expensive EEGNet. While this work focuses on the detection of attention using epoched EEG data, the experiment can also be used to study working memory [1]. Indeed, the complete sequence EEG data should permit an investigation regarding working memory and whether it is influenced by auditory and/or rhythmic support.

## Figures and Tables

**Table 1:** An example of a sequence of stimuli shown to the subject, with the targets in black and distractors in gray. In conditions with rhythm (Con2 and Con4), this sequence was preceded with five empty circles to induce the rhythm. The subject was expected to report the target digits and ignore the distractors.

Sequence	Targets	Distractors
③⑨②④③③◯①②⑧	39432	23 18

**Table 2:** Overview of the methods that were used in this study, together with their original target domain and methodology.

Model	Target Domain	Methodology
xDAWN + RG [29]	EEG	feature-based
MiniRocket [40]	time series	feature-based
Rocket [39]	time series	feature-based
EEGNet [33]	EEG	end-to-end

**Table 3:** Classifier test performance for individual subject models, averaged across the 42 subjects. The best performances are indicated in bold.

Model	Accuracy	F1 Score	AUC
xDAWN + RG	0.76±0.04	0.76±0.04	0.84±0.05
MiniRocket	0.69±0.05	0.70±0.05	0.76±0.06
Rocket	0.72±0.05	0.72±0.05	0.79±0.06
EEGNet	0.76±0.04	0.76±0.04	0.83±0.04

**Table 4:** Classifier performance for cross-subject models. Every subject was used as a test subject once; we report the average across all test sets. The best performances are indicated in bold.

Model	Accuracy	F1 Score	AUC
xDAWN + RG	0.73±0.04	0.72±0.06	0.81±0.05
MiniRocket	0.69±0.04	0.69±0.05	0.76±0.05
Rocket	0.72±0.05	0.71±0.06	0.79±0.05
EEGNet	0.76±0.04	0.76±0.05	0.84±0.05

**Table 5:** Drop in performance, calculated by subtracting the test performance of cross-subject models from that of individual subject models. The best performances are indicated in bold.

Model	Accuracy	F1 Score	AUC
xDAWN + RG	0.03 ± 0.02	0.04 ± 0.04	0.03 ± 0.02
MiniRocket	**0.00 ± 0.03**	0.01 ± 0.03	−0.00 ± 0.03
Rocket	**0.00 ± 0.02**	0.01 ± 0.04	−0.00 ± 0.03
EEGNet	**0.00 ± 0.02**	**0.00 ± 0.02**	**−0.01 ± 0.03**

**Table 6:** The test accuracies of the CS EEGNet model for the different conditions.

Condition	Accuracy
Con1	0.74
Con2	0.71
Con3	0.81
Con4	0.77

## Data Availability

Publicly available datasets were analyzed in this study. The behavioral data can be found at: https://osf.io/ntmy8/?view_only=88d951c394c7481dba00a1497d64797f, accessed on 17 October 2023. The preprocessed EEG data are openly available from Figshare at https://doi.org/10.6084/m9.figshare.24278887, accessed on 17 October 2023, reference number [79]. The raw EEG data will be released later as part of a separate publication.

## Abbreviations

The following abbreviations are used in this manuscript:

AI	Artificial intelligence
ALS	Amyotrophic lateral sclerosis
AUC	Area under the curve
BCI	Brain–computer interface
CNN	Convolutional neural network
CPU	Central processing unit
CS	Cross-subject
EEG	Electroencephalography
ERP	Event-related potential
FN	False negative
FP	False positive
GPU	Graphics processing unit
ICA	Independent component analysis
IS	Individual subject
LIME	Local interpretable model-agnostic explanations
LOO	Leave-one-out
ML	Machine learning
RG	Riemannian geometry
ROC AUC	Area under the receiver operating characteristic curve
TN	True negative
TP	True positive
xAI	Explainable AI

## Appendix A. Package Versions

**Table A1:** The versions of the Python packages used in the project.

Package	Version	Reference
python	3.9.13	[80]
MNE	1.2.1	[55]
pyriemann	0.3	[67]
torch	1.13.1	[81]
sktime	0.17.1	[71]
scikit-learn	1.1.3	[68]

## References

[1] De Winne J., Devos P., Leman M., Botteldooren D. (2022). With No Attention Specifically Directed to It, Rhythmic Sound Does Not Automatically Facilitate Visual Task Performance. Front. Psychol..

[2] Van der Burg E., Olivers C.N., Bronkhorst A.W., Theeuwes J. (2008). Pip and Pop: Nonspatial Auditory Signals Improve Spatial Visual Search. J. Exp. Psychol. Hum. Percept. Perform..

[3] Luck S.J., Woodman G.F., Vogel E.K. (2000). Event-related potential studies of attention. Trends Cogn. Sci..

[4] Woodman G.F. (2010). A brief introduction to the use of event-related potentials in studies of perception and attention. Atten. Percept. Psychophys.

[5] Näätänen R. (1975). Selective attention and evoked potentials in humans—A critical review. Biol. Psychol..

[6] Näätänen R., Gaillard A., Mäntysalo S. (1978). Early selective-attention effect on evoked potential reinterpreted. Acta Psychol..

[7] Näätänen R. (1990). The role of attention in auditory information processing as revealed by event-related potentials and other brain measures of cognitive function. Behav. Brain Sci..

[8] Duncan C.C., Barry R.J., Connolly J.F., Fischer C., Michie P.T., Näätänen R., Polich J., Reinvang I., Van Petten C. (2009). Event-related potentials in clinical research: Guidelines for eliciting, recording, and quantifying mismatch negativity, P300, and N400. Clin. Neurophysiol..

[9] Johnson R. (1988). The amplitude of the P300 component of the event-related potential: Review and synthesis. Adv. Psychophysiol..

[10] Gray H.M., Ambady N., Lowenthal W.T., Deldin P. (2004). P300 as an index of attention to self-relevant stimuli. J. Exp. Soc. Psychol..

[11] Riccio A., Simione L., Schettini F., Pizzimenti A., Inghilleri M., Belardinelli M.O., Mattia D., Cincotti F. (2013). Attention and P300-based BCI performance in people with amyotrophic lateral sclerosis. Front. Hum. Neurosci..

[12] Scharinger C., Soutschek A., Schubert T., Gerjets P. (2017). Comparison of the working memory load in N-back and working memory span tasks by means of EEG frequency band power and P300 amplitude. Front. Hum. Neurosci..

[13] Picton T.W. (1992). The P300 wave of the human event-related potential. J. Clin. Neurophysiol..

[14] Sutton S., Braren M., Zubin J., John E.R. (1965). Evoked-Potential Correlates of Stimulus Uncertainty. Science.

[15] Polich J. (2007). Updating P300: An Integrative Theory of P3a and P3b. Clin. Neurophysiol. Off. J. Int. Fed. Clin. Neurophysiol..

[16] Polich J. (2003). Detection of Change: Event-Related Potential and fMRI Findings.

[17] Donchin E. (1981). Surprise!… Surprise?. Psychophysiology.

[18] Nicolas-Alonso L.F., Gomez-Gil J. (2012). Brain Computer Interfaces, a Review. Sensors.

[19] Mak J.N., Wolpaw J.R. (2009). Clinical Applications of Brain—Computer Interfaces: Current State and Future Prospects. IEEE Rev. Biomed. Eng..

[20] Mak J.N., Arbel Y., Minett J.W., McCane L.M., Yuksel B., Ryan D., Thompson D., Bianchi L., Erdogmus D. (2011). Optimizing the P300-based brain–computer interface: Current status, limitations and future directions. J. Neural Eng..

[21] Guy V., Soriani M.H., Bruno M., Papadopoulo T., Desnuelle C., Clerc M. (2018). Brain computer interface with the P300 speller: Usability for disabled people with amyotrophic lateral sclerosis. Ann. Phys. Rehabil. Med..

[22] Kaper M., Meinicke P., Grossekathoefer U., Lingner T., Ritter H. (2004). BCI competition 2003—Data set IIb: Support vector machines for the P300 speller paradigm. IEEE Trans. Biomed. Eng..

[23] Okahara Y., Takano K., Komori T., Nagao M., Iwadate Y., Kansaku K. (2017). Operation of a P300-based brain-computer interface by patients with spinocerebellar ataxia. Clin. Neurophysiol. Pract..

[24] Aydin E.A., Bay O.F., Guler I. (2018). P300-based asynchronous brain computer interface for environmental control system. IEEE J. Biomed. Health Inform..

[25] Masud U., Baig M.I., Akram F., Kim T.S. A P300 brain computer interface based intelligent home control system using a random forest classifier. Proceedings of the 2017 IEEE Symposium Series on Computational Intelligence (SSCI) 2017.

[26] Ikegami S., Takano K., Kondo K., Saeki N., Kansaku K. (2014). A region-based two-step P300-based brain–computer interface for patients with amyotrophic lateral sclerosis. Clin. Neurophysiol..

[27] Guger C., Daban S., Sellers E., Holzner C., Krausz G., Carabalona R., Gramatica F., Edlinger G. (2009). How many people are able to control a P300-based brain–computer interface (BCI)?. Neurosci. Lett..

[28] Aggarwal S., Chugh N. (2022). Review of Machine Learning Techniques for EEG Based Brain Computer Interface. Arch. Comput. Methods Eng..

[29] Rivet B., Souloumiac A., Attina V., Gibert G. (2009). xDAWN Algorithm to Enhance Evoked Potentials: Application to Brain-Computer Interface. IEEE Trans. Biomed. Eng..

[30] Congedo M., Barachant A., Bhatia R. (2017). Riemannian geometry for EEG-based brain-computer interfaces; a primer and a review. Brain-Comput. Interfaces.

[31] Barachant A., Bonnet S., Congedo M., Jutten C. (2012). Multiclass brain-computer interface classification by Riemannian geometry. IEEE Trans. Biomed. Eng..

[32] Barachant A., Bonnet S., Congedo M., Jutten C. (2013). Classification of covariance matrices using a Riemannian-based kernel for BCI applications. Neurocomputing.

[33] Lawhern V.J., Solon A.J., Waytowich N.R., Gordon S.M., Hung C.P., Lance B.J. (2018). EEGNet: A compact convolutional neural network for EEG-based brain-computer interfaces. J. Neural Eng..

[34] Pereira A.E., Padden D., Jantz J.J., Lin K., Alcaide-Aguirre R.E. (2018). Cross-Subject EEG Event-Related Potential Classification for Brain-Computer Interfaces Using Residual Networks. HAL Open Sci..

[35] Zhang H., Wang Z., Yu Y., Yin H., Chen C., Wang H. (2022). An improved EEGNet for single-trial EEG classification in rapid serial visual presentation task. Brain Sci. Adv..

[36] Shamsi F., Haddad A., Najafizadeh L., Zang B., Lin Y., Liu Z., Awwad Shiekh Hasan B., Gan J.Q. (2019). Deep learning for electroencephalogram (EEG) classification tasks: A review. J. Neural Eng..

[37] Kulasingham J.P., Vibujithan V., De Silva A.C. Deep belief networks and stacked autoencoders for the P300 Guilty Knowledge Test. Proceedings of the IECBES 2016—IEEE-EMBS Conference on Biomedical Engineering and Sciences.

[38] Miao Z., Zhao M., Zhang X., Ming D. (2023). LMDA-Net:A lightweight multi-dimensional attention network for general EEG-based brain-computer interfaces and interpretability. NeuroImage.

[39] Dempster A., Petitjean F., Webb G.I. (2020). ROCKET: Exceptionally fast and accurate time series classification using random convolutional kernels. Data Min. Knowl. Discov..

[40] Dempster A., Schmidt D.F., Webb G.I. MINIROCKET: A Very Fast (Almost) Deterministic Transform for Time Series Classification. Proceedings of the ACM SIGKDD International Conference on Knowledge Discovery and Data Mining.

[41] Dutta K.K. Multi-class time series classification of EEG signals with recurrent neural networks. Proceedings of the 9th International Conference On Cloud Computing, Data Science and Engineering, Confluence 2019.

[42] Gordon S.M., Jaswa M., Solon A.J., Lawhern V.J. Real world BCI: Cross-domain learning and practical applications. Proceedings of the BCIforReal 2017—The 2017 ACM Workshop on An Application-Oriented Approach to BCI out of the Laboratory, co-Located with IUI 2017.

[43] Wu D. (2017). Online and Offline Domain Adaptation for Reducing BCI Calibration Effort. IEEE Trans. Hum.-Mach. Syst..

[44] Ma B.Q., Li H., Zheng W.L., Lu B.L. (2019). Reducing the subject variability of eeg signals with adversarial domain generalization. Neural Information Processing: 26th International Conference, ICONIP 2019, Sydney, Australia, 12–15 December 2019.

[45] Wu D., Xu Y., Lu B.L. (2020). Transfer learning for EEG-based brain–computer interfaces: A review of progress made since 2016. IEEE Trans. Cogn. Dev. Syst..

[46] Morioka H., Kanemura A., Hirayama J.i., Shikauchi M., Ogawa T., Ikeda S., Kawanabe M., Ishii S. (2015). Learning a common dictionary for subject-transfer decoding with resting calibration. NeuroImage.

[47] Linardatos P., Papastefanopoulos V., Kotsiantis S. (2020). Explainable AI: A Review of Machine Learning Interpretability Methods. Entropy.

[48] Ribeiro M.T., Singh S., Guestrin C. “Why should I trust you?” Explaining the predictions of any classifier. Proceedings of the ACM SIGKDD International Conference on Knowledge Discovery and Data Mining.

[49] Islam M.S., Hussain I., Rahman M.M., Park S.J., Hossain M.A. (2022). Explainable Artificial Intelligence Model for Stroke Prediction Using EEG Signal. Sensors.

[50] Shrikumar A., Greenside P., Kundaje A. Learning Important Features Through Propagating Activation Differences. Proceedings of the International Conference on Machine Learning.

[51] Gabeff V., Teijeiro T., Zapater M., Cammoun L., Rheims S., Ryvlin P., Atienza D. (2021). Interpreting deep learning models for epileptic seizure detection on EEG signals. Artif. Intell. Med..

[52] Simonyan K., Vedaldi A., Zisserman A. (2013). Deep Inside Convolutional Networks: Visualising Image Classification Models and Saliency Maps. arXiv.

[53] Farahat A., Reichert C., Sweeney-Reed C.M., Hinrichs H. (2019). Convolutional neural networks for decoding of covert attention focus and saliency maps for EEG feature visualization. J. Neural Eng..

[54] Aslan Z., Akin M. (2022). A deep learning approach in automated detection of schizophrenia using scalogram images of EEG signals. Phys. Eng. Sci. Med..

[55] Gramfort A., Luessi M., Larson E., Engemann D.A., Strohmeier D., Brodbeck C., Goj R., Jas M., Brooks T., Parkkonen L. (2013). MEG and EEG data analysis with MNE-Python. Front. Neurosci..

[56] Cohen M.X. (2014). Analyzing Neural Time Series Data: Theory and Practice.

[57] Patel S.H., Azzam P.N. (2005). Characterization of N200 and P300: Selected Studies of the Event-Related Potential. Int. J. Med Sci..

[58] Demiralp T., Ademoglu A., Istefanopulos Y., Başar-Eroglu C., Başar E. (2001). Wavelet analysis of oddball P300. Int. J. Psychophysiol..

[59] Geraghty J., Schoettle G. Single-Subject vs. Cross-Subject Motor Imagery Models. Proceedings of the International Conference on Human-Computer Interaction.

[60] Kwon O.Y., Lee M.H., Guan C., Lee S.W. (2020). Subject-Independent Brain-Computer Interfaces Based on Deep Convolutional Neural Networks. IEEE Trans. Neural Networks Learn. Syst..

[61] Ghane P., Zarnaghinaghsh N., Braga-Neto U. Comparison of Classification Algorithms Towards Subject-Specific and Subject-Independent BCI. Proceedings of the 9th IEEE International Winter Conference on Brain-Computer Interface, BCI 2021.

[62] Fazli S., Grozea C., Danóczy M., Popescu F., Blankertz B., Müller K.R. (2009). Subject independent EEG-based BCI decoding. Adv. Neural Inf. Process. Syst..

[63] Wold S., Esbensen K., Geladi P. (1987). Principal component analysis. Chemom. Intell. Lab. Syst..

[64] Pahuja S.K., Veer K. (2022). Recent Approaches on Classification and Feature Extraction of EEG Signal: A Review. Robotica.

[65] Barachant A., Congedo M. (2014). A plug&play P300 BCI using information geometry. arXiv.

[66] Congedo M., Barachant A., Andreev A. (2013). A new generation of brain-computer interface based on riemannian geometry. arXiv.

[67] Barachant A., Barthélemy Q., King J.R., Gramfort A., Chevallier S., Rodrigues P.L.C., Olivetti E., Goncharenko V., vom Berg G.W., Reguig G. (2022). pyRiemann/pyRiemann: V0.3. https://zenodo.org/records/7547583.

[68] Pedregosa F., Varoquaux G., Gramfort A., Michel V., Thirion B., Grisel O., Blondel M., Prettenhofer P., Weiss R., Dubourg V. (2011). Scikit-learn: Machine Learning in Python. J. Mach. Learn. Res..

[69] Zancanaro A., Cisotto G., Paulo J.R., Pires G., Nunes U.J. CNN-based Approaches For Cross-Subject Classification in Motor Imagery: From the state-of-the-art to DynamicNet. Proceedings of the 2021 IEEE Conference on Computational Intelligence in Bioinformatics and Computational Biology, CIBCB 2021.

[70] Dau H.A., Bagnall A., Kamgar K., Yeh C.C.M., Zhu Y., Gharghabi S., Ratanamahatana C.A., Keogh E. (2018). The UCR Time Series Archive. IEEE/CAA J. Autom. Sin..

[71] Löning M., Bagnall A., Ganesh S., Kazakov V., Lines J., Király F.J. (2019). sktime: A unified interface for machine learning with time series. arXiv.

[72] Roy Y., Banville H., Albuquerque I., Gramfort A., Falk T.H., Faubert J. (2019). Deep learning-based electroencephalography analysis: A systematic review. J. Neural Eng..

[73] Reinhart R.M., Mathalon D.H., Roach B.J., Ford J.M. (2011). Relationships between pre-stimulus gamma power and subsequent P300 and reaction time breakdown in schizophrenia. Int. J. Psychophysiol..

[74] Li F., Tao Q., Peng W., Zhang T., Si Y., Zhang Y., Yi C., Biswal B., Yao D., Xu P. (2020). Inter-subject P300 variability relates to the efficiency of brain networks reconfigured from resting- to task-state: Evidence from a simultaneous event-related EEG-fMRI study. NeuroImage.

[75] Al-Nafjan A., Aldayel M. (2022). Predict Students’ Attention in Online Learning Using EEG Data. Sustainability.

[76] Hu B., Li X., Sun S., Ratcliffe M. (2018). Attention Recognition in EEG-Based Affective Learning Research Using CFS+KNN Algorithm. IEEE/ACM Trans. Comput. Biol. Bioinform..

[77] Li F., Xia Y., Wang F., Zhang D., Li X., He F. (2020). Transfer Learning Algorithm of P300-EEG Signal Based on XDAWN Spatial Filter and Riemannian Geometry Classifier. Appl. Sci..

[78] Gayraud N.T., Rakotomamonjy A., Clerc M. Optimal transport applied to transfer learning for P300 detection. Proceedings of the BCI 2017-7th Graz Brain-Computer Interface Conference.

[79] Mortier S., De Winne J., Sun P., Vanransbeeck W., Turkes R., Yuan Z., Verdonck T., Leman M., Devos P., Botteldooren D. (2023). WithMe Preprocessed Dataset. https://figshare.com/articles/dataset/WithMe_preprocessed_dataset/24278887/1.

[80] Van Rossum G., Drake F.L. (2009). Python 3 Reference Manual.

[81] Paszke A., Gross S., Massa F., Lerer A., Bradbury J., Chanan G., Killeen T., Lin Z., Gimelshein N., Antiga L. (2019). Pytorch: An imperative style, high-performance deep learning library. Adv. Neural Inf. Process. Syst..

